# Cilostazol induced migraine does not respond to sumatriptan in a double blind trial

**DOI:** 10.1186/s10194-018-0841-7

**Published:** 2018-02-02

**Authors:** Katrine Falkenberg, Bára Óladóttir á Dunga, Song Guo, Messoud Ashina, Jes Olesen

**Affiliations:** Danish Headache Centre and Department of Neurology, University of Copenhagen, Rigshospitalet Glostrup, Copenhagen, DK-2600, Glostrup, Denmark

**Keywords:** Headache, Migraine, Pain, Phosphodiesterase type 3, Human migraine model

## Abstract

**Background:**

Cilostazol is an inhibitor of phosphodiesterase 3 and thus causes accumulation of cAMP. It induces migraine-like attacks in migraine patients. Whether the cilostazol model responds to sumatriptan in migraine patients and therefore is valid for testing of future anti-migraine medications has never been investigated.

**Methods:**

In a cross-over study, 30 patients received cilostazol (200 mg p.o.) on two separate days each day followed by oral self-administered placebo or sumatriptan 50 mg. We recorded headache characteristics and associated symptoms using a questionnaire. The 30 participants were asked to subsequently treat their spontaneous attacks with sumatriptan (50 mg) or placebo in a double-blind cross-over design and 15 participants did so.

**Results:**

Cilostazol induced headache with some migraine characteristics in all participants; 18 patients on the sumatriptan day and 19 patients on the placebo day fulfilled criteria for a migraine-like attack. The difference in median headache intensity between sumatriptan and placebo at 2 h was not significant (*p* = 0.09), but it was at 4 h (*p* = 0.017). During spontaneous attacks, the difference between placebo and sumatriptan was not significant at 2 h (*p* = 0.26), but it was highly significant at 4 h (*p* = 0.006).

**Conclusion:**

The cilostazol model in migraine patients could not be validated by a sufficient sumatriptan response. The model may perhaps respond to new drugs that act intracellularly or directly on ion channels.

**Trial registration:**

The study is registered on clinicaltrials.gov (NCT02486276)

**Electronic supplementary material:**

The online version of this article (10.1186/s10194-018-0841-7) contains supplementary material, which is available to authorized users.

## Background

The current treatments for migraine are not satisfactory and there remains a great need for new acute and prophylactic anti-migraine drugs. Testing new drugs in spontaneous migraine is cumbersome and it is done outside of the hospital which makes additional study of pharmacokinetic and -dynamics difficult. A valid experimental model could test new drugs under standardized circumstances and in a short period.

Since healthy volunteers are easiest to recruit and tablets are the preferred mode of administration of migraine treatment, we first tried to develop and validate a model in healthy subjects using isosorbide-5-mononitrate (5-ISMN) [1] and cilostazol [2] as the headache inducing substances. In both trials, we used sumatriptan tablets to validate the model. Sumatriptan tablets had no effect on 5-ISMN induced headache [1], and only a trend toward efficacy on cilostazol induced headache in healthy volunteers [2]. Consequently, we now try to develop a model in patients with migraine using cilostazol as the headache inducing substance and sumatriptan to validate the model. Previous studies have shown that cilostazol causes migraine-like attacks in 86% of patients without aura [3, 4]. Cilostazol is an inhibitor of phosphodiesterase 3 (PDE3) which breaks down cyclic adenylate monophosphate (cAMP). When break down is inhibited, cAMP accumulates leading to general vasodilatation. Since cAMP accumulation is the only effect of cilostazol, cAMP must be the cause of headache/migraine-like attacks after cilostazol administration [3]. Whether the headache is a consequence of the vasodilatation or due to other effects of cAMP is not known. Whether the cilostazol model in migraine patients can be used to test current and future anti-migraine medications has never been validated. In a previous study we induced migraine-like attacks in MO patients with cilostazol and the patients treated the induced migraine attacks with a triptan in an open and uncontrolled fashion [3]. Patients responded well but at a late time after the provocation and perhaps due to the placebo effect. It is therefore necessary to test the efficacy of sumatriptan against cilostazol induced headache/migraine in a double-blind, cross-over study.

## Methods

The method has previously been described in two studies on healthy volunteers by the authors [1, 2].

### Participants

Thirty patients with migraine without aura were included. 23 of the patients were self-reported triptan responders and 7 were triptan-naive. Inclusion criteria were: Patients fulfilling IHS criteria for migraine without aura of both sexes, aged 18–60 years and weighing 45–95 kg. Females were requested to use effective contraception.

Exclusion criteria were: Patients fulfilling any other type of headache than MO (except episodic tension-type headache < 1 day per week), self-reported triptan non-responders, serious somatic or psychiatric disease, pregnancy, and intake of daily medication (except oral contraceptives).

One participant dropped out for personal reasons. She was replaced with a new participant.

After completion of the study and with the knowledge of a partly negative response of sumatriptan to cilostazol headache, we wanted to be sure that the response was not due to lack of efficacy of sumatriptan in the study population. Therefore, we contacted all 30 participants and invited them to treat their spontaneous attacks with sumatriptan in a double-blind fashion. 2 patients did not suffer from migraine anymore, 2 patients were pregnant, 2 patients never responded to our request and 9 patients declined to participate. Fifteen patients accepted to treat their spontaneous attacks, and this post ad-hoc analysis was thus slightly underpowered.

### Design

We conducted a double-blinded, randomized, balanced, placebo-controlled, cross-over study in which cilostazol 200 mg was given orally on two separate days, five days or more apart. The provocation was both days followed by oral self- administrated placebo or sumatriptan 50 mg. The 15 patients, who subsequently treated their spontaneous attacks in a double-blind fashion, did so at least one month after the provocation study.

The central pharmacy of the Capital Region of Copenhagen performed the randomization of the experimental drug in a balanced fashion. The randomization code did not leave the hospital during the study and was not available to the investigators until after termination of the study. We did not break the code until data management took place.

### Standard protocol approvals

All participants gave written, informed consent to participate in the study. The study was approved by the Ethics Committee of Copenhagen (H-8-2014-009), the Danish Data Protection Agency, and the Danish Medicines Agency. The study is registered on clinicaltrials.gov (NCT02486276) and was conducted according to the Helsinki II declaration of 1964, as revised in 2008.

All participants were enrolled via the website forsøgsperson.dk [5], through the Danish Headache Center or through patient organizations.

### Study procedure

The patients had to be headache free 48 h prior to the study and not to have taken any type of painkillers 12 h before beginning of the study. A pregnancy test was taken at the beginning of each study day on all fertile female participants. All participants had two separate study days at least five days apart. They arrived non-fasting at the clinic between 8:00 a.m. and 12:00 a.m. Full medical history, physical examination, electrocardiography (ECG), vital signs and baseline headache were collected at arrival. All participants received cilostazol 200 mg orally on both study days and went home immediately after. When the participants reached headache intensity 4 on the numerical rating scale (NRS) or six hours after cilostazol, the treatment (placebo or sumatriptan) was taken. All patients were thoroughly instructed about time of medication intake and noted time of intake in a questionnaire so the authors could ensure that treatment was taken appropriately. Time of treatment was chosen as a trade-off between treating early enough (sumatriptan is most effective when taking early in an attack) and not treating before migraine mechanisms were activated. Also, we needed the patients to have a measurable degree of headache before treatment. In case of severe headache not responding to the experimental treatment, the participants were allowed rescue with their usual anti-migraine medication or nonsteroidal anti-inflammatory drugs (NSAIDs) but not before two hours after placebo or sumatriptan. According to the patients’ report, no one took rescue medication before 2 h after treatment (see Table 2) and we have to believe on the patients’ report.

During the study, an emergency phone was always open where patients could call if they experienced severe headaches or discomfort.

The 15 patients, who treated their spontaneous attacks also took the treatment (sumatriptan or placebo) when headache intensity was 4 on NRS. The participants were also allowed rescue with their usual anti-migraine treatment two hours after the experimental drug, if the headache did not respond to the experimental drug.

### Headache parameters

Headache parameters and accompanying symptoms were recorded by the investigator at baseline on a headache questionnaire. Afterward headache intensity, characteristics (unilateral/bilateral, quality and aggravation by physical activity), accompanying symptoms (nausea/vomiting, phono- and photophobia) and side effects were scored on a self-administered questionnaire. The patients had to fill out the questionnaire every 30 min the first six hours after cilostazol and thereafter every hour until 12 h after cilostazol. The intensity was scored on a Numerical Rating Scale (NRS) from 0 to 10, 1 representing a very mild headache (including feeling of pressing or pulsation), 5 a headache of medium severity and 10 the worst possible headache (10). All patients had a thorough instruction for the questionnaire. The rational for a self-administered questionnaire is feasibility of the study. Few patients would accept staying at the hospital for over 12 h and especially not while having a migraine attack. So for the sake of the patients, they were allowed to treat at home.

One patient went to bed at 11 h. To make it possible to plot the 0-12 h curve, the missing data at 12 h were filled in using last observation carried forward.

When the patients treated their spontaneous migraine attacks, they were instructed to fill out the questionnaire when they felt an attack and to keep doing so for at least 6 h after the experimental drug.

The following criterion was used for a migraine-like attack induced 0–12 h after administration of cilostazol:

Headache fulfilling criteria C and D for migraine without aura according to the IHS criteria [6].

C. C. Headache has at least two of the following characteristics:

- Unilateral location
- Pulsating quality
- Moderate or severe pain intensity (moderate to severe pain intensity is considered ≥4 on NRS)
- Aggravation by cough or causing avoidance of routine physical activity

D. D. During headache at least one of the following:

- Nausea and/or vomiting
- Photophobia and phonophobia.

### Statistical analysis

Calculation of sample size was based on the detection of a difference in headache intensity between two experimental days, at 5% significance with 90% power. We estimated that placebo had an effect on 20% and sumatriptan had 60% effect. Standard deviation was estimated based on previous data. The correlation between the two days was estimated conservatively at 0.5. We also assumed no carry-over effect. We calculated that at least 18 participants should complete both experimental days. Due to uncertainty regarding these assumptions we decided to include 30 participants. The area under the curve (AUC) for headache score was used as a summary measure for analyzing differences between the groups and was calculated according to the trapezium rule (12). Pain-freedom is the recommended primary outcome parameter in clinical trials, but in experimental studies it is more powerful to score pain on Numerical Rating Scale (NRS) 0–10 and use median headache score as outcome parameter. Even though the effect of triptans is higher at 4 h than at 2 h, clinical migraine trials always study the effect of pain relieve after 2 h. Therefore, our primary endpoints were (1) difference in median headache intensity between sumatriptan and placebo at two hours after treatment, and (2) difference in AUC 0-4 h between the two experimental days. Secondary end-points were difference in pain intensity difference (PID) between the sumatriptan day and the placebo day, (PID is the difference in pain intensity at the various time points versus baseline. This will take headache score at treatment time into account (headache score varies from 0 to 6 with a median headache score at 4)), difference in median peak headache score, difference in median headache intensity between the two treatments at 4 h, AUC 0-2 h after sumatriptan/placebo and accompanying symptoms as nausea, photo- and photophobia.

Headache intensity scores are presented as medians (range). Differences in median headache scores and AUC for headache scores were tested using the Wilcoxon signed rank test. Difference in pain intensity difference between the sumatriptan day and the placebo day were tested using Mann-Whitney U test. The incidence of headache and associated symptoms were analyzed as binary categorical data with McNemar’s test. Age and weigh are presented as means. We did not correct for multiple testing. All analyses were performed with SPSS for Windows 11.5 (Chicago, IL, USA), or GraphPad Prism version 7.0. A *p* < 0.05 was considered significant.

## Results

**Fig. 1:**
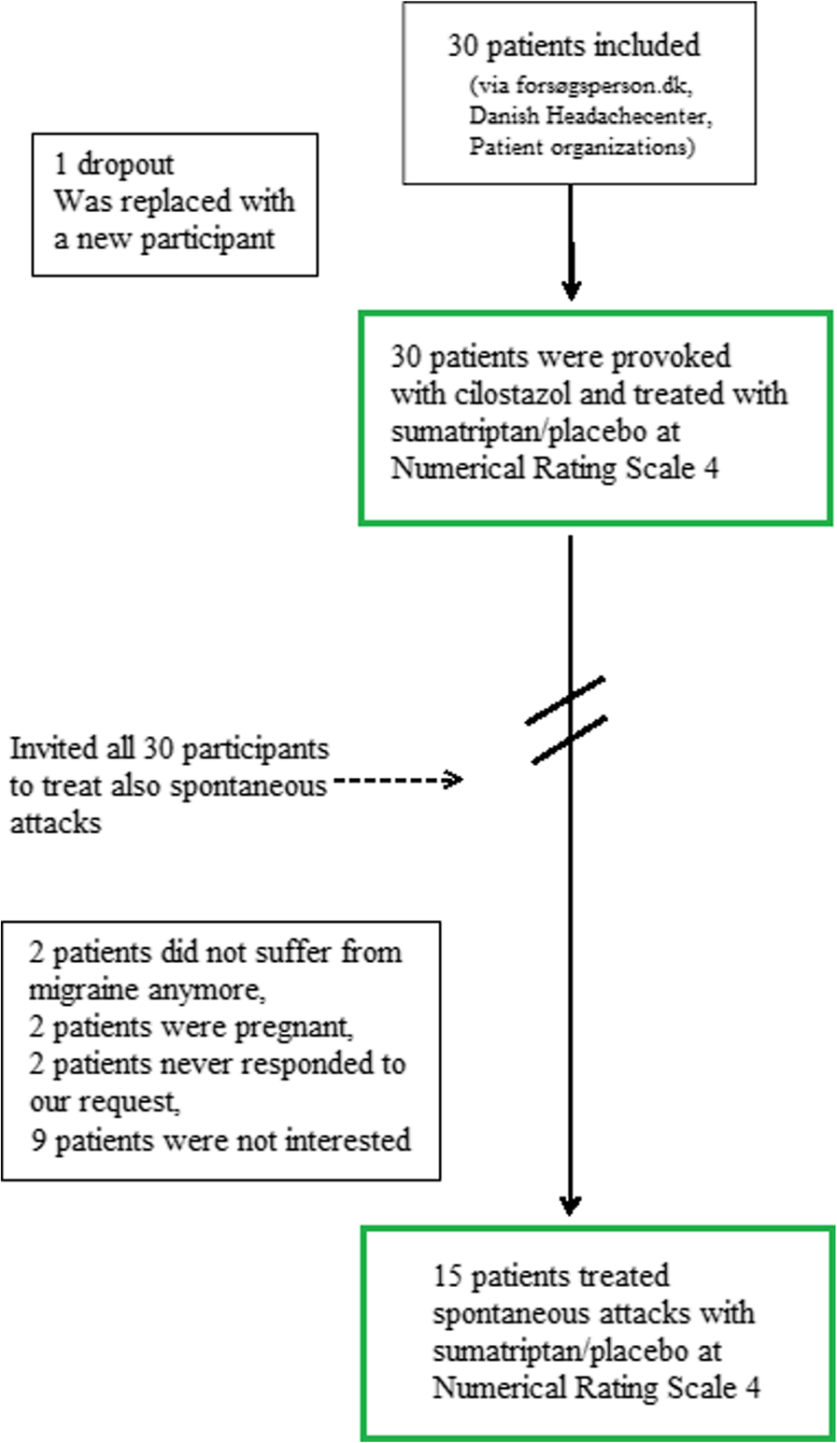
Study enrolment

Thirty patients with migraine without aura (22F, 8 M) completed both days of the provocation study. Mean age was 34.4 years (range 21–59 years) and mean weight was 69.8 kg (range 47–95 kg). Mean attack frequency per month was 3.9 (range 1–10). Fifteen of the 30 patients reported a first degree relative with migraine. All 30 patients were asked, but only 15 patients subsequently treated their spontaneous migraine attacks with sumatriptan and placebo in a double-blind cross-over fashion (Fig. 1).

### Cilostazol induced headache

**Fig. 2:**
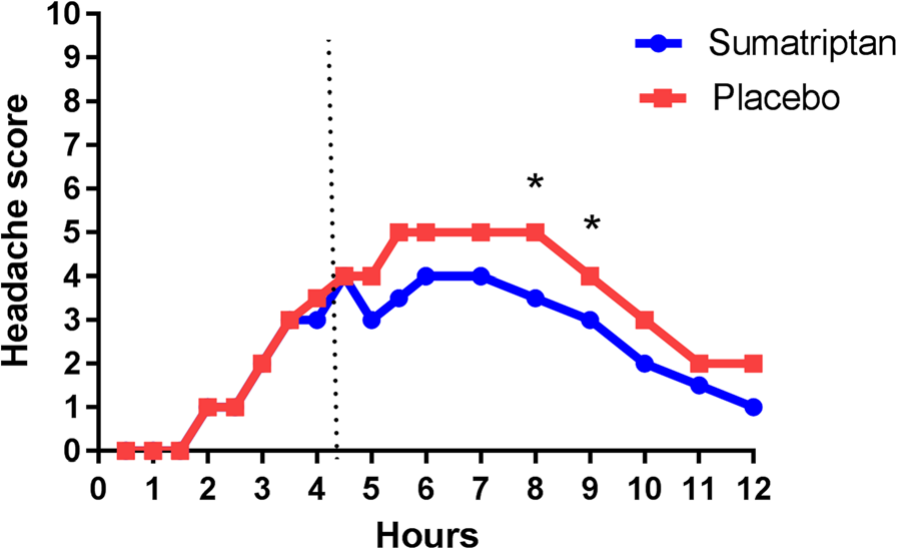
Median headache score after cilostazol. Median headache score 0-12 h after cilostazol on the two treatment days. Median time to treatment was 4.5 h on the sumatriptan day and 4.25 h on the placebo day illustrated by the dotted line. The difference between the two treatment days is significant at 4 h (*p* = 0.017) and 5 h (*p* = 0.028) after treatment

Cilostazol induced headache in all patients on both days (NRS range 1–10). The headache had several migraine-like features (summarized in Table 1); 23 patients (77%) fulfilled criteria for a migraine-like attack on either one or both provocation days, 18 patients (60%) on the sumatriptan day and 19 (63%) on the placebo day. Median time to migraine attack was 4 h (range 2-8 h) on the sumatriptan day and 5 h (range 0.5-9 h) on the placebo day. Headache characteristics and associated symptoms on the two experimental days are presented in Table 1. Characteristics of the headache for each subject are presented in Table 2. Median headache score 0-12 h after cilostazol for the two treatment groups is illustrated in Fig. 2. Note that the median headache score is reproducible between the two days until time of treatment. The figure also illustrates that median headache score at treatment was 4 (range 0–6) on both days and median time to treatment was 4.5 h (range 2-6 h) on the sumatriptan day and 4.25 h (range 1.5-6 h) on the placebo day.

**Table 1:** Clinical characteristics (our secondary end-point) of headache and associated symptoms after cilostazol

	Sumatriptan (*n* = 30)	Placebo (*n* = 30)	*p*-value ^a^
Number of participants reporting headache	30	30	1.00
(range 1–10 on NRS)			
Median peak headache score (range)	5 (1–10)	7 (1–10)	0.03^b^
*No. of participants with*			
Unilateral location	15	16	1.00
Throbbing headache	19	21	0.63
Aggravation by physical activity	26	27	1.00
Nausea	17 (2^c^)	17 (3^c^)	1.00
Photophobia	14	17	0.25
Phonophobia	10	14	0.29
Rescue medication	25	29	0.13
Migraine-like attack	18	19	1.00

^a^McNemar’s test

^b^Wilcoxon signed rank test

^c^Subject vomited

**Table 2:** Characteristics of the headache for each subject

Subject	Peak headache	Characteristics^a^	Associated symptoms^b^	Migraine-like attack^d^	Rescue treatment (hours)
1: Suma	5 (5.5 h)	+/+/+	−/+/+	Yes (5 h)	Bonyl (7 h)
1: Placebo	5 (5.5 h)	+/+/+	−/+/+	Yes (4 h)	Bonyl (6 h)
2: Suma	7 (7 h)	−/−/+	−/−/−	No	Bonyl (9 h)
2: Placebo	8 (3 h)	+/+/+	+/+/+	Yes (2 h)	Bonyl (8 h)
3: Suma	5 (7)	−/−/+	−/−/−	No	Bonyl (9 h)
3: Placebo	10 (10 h)	−/−/+	+/+/+^c^	Yes (8 h)	Bonyl (7.5 h) + Treo (12.5 h)
4: Suma	4 (5.5)	−/+/+	−/+/−	No	Bonyl (10 h)
4: Placebo	8 (5.5 h)	−/+/+	+/+/+^c^	Yes (5.5 h)	Bonyl (5.5 h) - Threw it up
5: Suma	8 (3.5 h)	−/−/+	+/−/−^c^	Yes (2.h)	Bonyl (4.5 h) + Ibuprofen & Para (6 h)
5: Placebo	7 (4.5 h)	−/−/+	+/−/−^c^	Yes (3.5 h)	Bonyl (5 h)
6: Suma	4 (4.5 h)	−/+/+	+/+/+	Yes (3.5 h)	Bonyl (5.5 h)
6: Placebo	7 (4.5 h)	+/+/+	+/+/+	Yes (3 h)	Bonyl (6 h)
7: Suma	9 (8 h)	+/+/+	+/+/+	Yes (3 h)	Bonyl (8 h)
7: Placebo	8 (11 h)	+/+/+	+/+/+	Yes (8 h)	Bonyl (12 h)
8: Suma	5 (4.5 h)	−/+/+	−/−/−	No	Bonyl (6 h)
8: Placebo	7 (6 h)	+/+/+	−/−/−	No	Zolmitriptan (6 h)
9: Suma	6 (6 h)	+/−/+	+/+/−	Yes (6 h)	Bonyl (8 h) + Sumatriptan (9.5 h)
9: Placebo	6 (5 h)	−/+/+	+/+/−	Yes (3.5 h)	Bonyl & Sumatriptan (4.5 h)
10: Suma	1 (2 h)	−/−/−	−/−/−	No	None
10: Placebo	1 (2 h)	−/−/−	−/−/−	No	None
11: Suma	5 (6 h)	+/+/+	+/−/−	Yes (6 h)	Sumatriptan (8 h)
11: Placebo	4 (5.5 h)	+/+/+	−/−/−	No	Bonyl (7 h) + Sumatriptan (11.5 h)
12: Suma	5 (3.5 h)	+/+/+	+/+/−	Yes (2.5 h)	Sumatriptan & Paracetamol (5 h)
12: Placebo	7 (3.5 h)	+/+/+	+/+/+	Yes (0.5 h)	Sumatriptan & Paracetamol (4 h) + Bonyl (5.5 h)
13: Suma	5 (4 h)	+/+/−	+/+/+	Yes (4 h)	None
13: Placebo	8 (5 h)	+/+/+	+/+/−	Yes (1 h)	Relpax (5 h)
14: Suma	4 (4.5 h)	+/+/+	+/+/−	Yes (4 h)	Bonyl (6 h) + Rizatriptan (7 h) + Treo (8 h)
14: Placebo	8 (7 h)	+/+/+	+/+/−	Yes (5 h)	Bonyl (7 h) + Rizatriptan (7.5 h) + Treo (9 h)
15: Suma	8 (5.5 h)	+/+/+	+/+/+	Yes (4.5 h)	Bonyl (6.5 h)
15: Placebo	5 (6 h)	−/+/−	+/+/−	Yes (6 h)	Bonyl (9 h)
16: Suma	5 (4.5 h)	+/+/+	+/−/+	Yes (4 h)	Bonyl (10.5 h)
16: Placebo	8 (8 h)	−/+/+	+/−/+	Yes (5 h)	Bonyl (7.5 h) + Sumatriptan (7.5 h)
17: Suma	7 (9 h)	−/−/+	+/−/−	Yes (8 h)	Bonyl (2 h) + Sumatriptan (11.5 h)
17: Placebo	8 (7 h)	−/−/+	−/−/−	No	Bonyl & Sumatriptan (6 h)
18: Suma	4 (6 h)	−/+/−	−/−/−	No	None
18: Placebo	7 (5.5 h)	−/+/+	−/+/−	No	Treo (5.5 h)
19: Suma	10 (3 h)	−/−/+	+/−/+	Yes (2.5 h)	Sumatriptan & Bonyl & Paracetamol (4 h)
19: Placebo	6 (5 h)	−/−/+	−/−/+	No	Bonyl&Para.& Suma (5.5 h) + Suma&Para. (7.5 h)
20: Suma	5 (8 h)	+/+/+	+/+/+^c^	Yes (5 h)	Bonyl & Sumatriptan (7 h) + Sumatriptan (9 h)
20: Placebo	6 (9 h)	+/+/+	+/+/+	Yes (5.5 h)	Bonyl & Sumatriptan (7.5 h) + Sumatriptan (9 h)
21: Suma	4 (4.5 h)	+/−/−	−/−/−	No	Sumatriptan (9 h)
21: Placebo	4 (5 h)	+/−/−	−/−/−	No	Sumatripan (8 h)
22: Suma	6 (10 h)	+/−/+	−/+/−	No	None
22: Placebo	8 (9 h)	+/+/+	+/+/+	Yes (9 h)	Sumatriptan (7 h)
23: Suma	4 (3.5 h)	+/+/+	−/−/−	No	None
23: Placebo	9 (7 h)	+/+/+	+/−/−	Yes (7 h)	Sumatriptan (6 h)
24: Suma	3 (6 h)	−/−/+	−/−/−	No	Paracetamol (9 h)
24: Placebo	4 (8 h)	−/−/+	−/−/−	No	Bonyl (8 h)
25: Suma	5 (3.5)	+/+/+	+/−/−	Yes (3.5 h)	Bonyl (5.5 h) + Zolmitriptan (6.5 h)
25: Placebo	7 (7 h)	+/+/+	+/−/−	Yes (4 h)	Bonyl (6 h)
26: Suma	8 (5 h)	−/+/+	−/+/−	No	Bonyl & Paracetamol (8 h)
26: Placebo	8 (6 h)	+/+/+	−/+/−	No	Bonyl (7.5 h)
27: Suma	9 (6 h)	−/+/+	+/+/+	Yes (2.5 h)	Sumatriptan (6 h) + Excedrin (7 h)
27: Placebo	7 (4.5 h)	+/+/+	−/+/+	Yes (4 h)	Excedrin (6.5 h) + Bonyl (7 h)
28: Suma	7 (5 h)	−/+/+	+/+/+	Yes (2.5 h)	Bonyl & Sumatriptan (5.5 h) + Sumatriptan (8.5 h)
28: Placebo	8 (7 h)	−/+/+	+/+/+	Yes (6 h)	Bonyl & Sumatriptan (6.5 h)
29: Suma	5 (9 h)	+/+/+	+/−/−	Yes (8 h)	Bonyl (8.5 h)
29: Placebo	2 (10 h)	−/−/+	−/−/−	No	Bonyl (8 h)
30: Suma	7 (8 h)	−/−/+	−/−/−	No	Bonyl (9 h)
30: Placebo	8 (8 h)	−/−/+	−/−/+	No	Dolol (9 h)

^a^Characteristics: Location/quality/aggravation

^b^Associated symptoms: Nausea/photophobia/phonophobia (^c^ = vomited)

^d^Fulfilled criteria for an experimental induced migraine-like attack (hours)

*Para* = Paracetamol, *Suma* = Sumatriptan

### Effect of sumatriptan on cilostazol induced headache

**Fig. 3:**
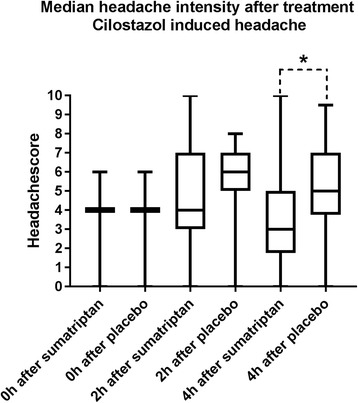
Median headache intensity after treatment of cilostazol induced headache. Median headache score at different time points after treatment of cilostazol induced headache. Difference in median headache intensity at 2 h: *p* = 0.09. Difference in median headache intensity at 4 h: *p* = 0.017

The difference in median headache intensity between sumatriptan and placebo at 2 h after treatment (our primary end-point), was not statistically significant (*p =* 0.09). At 2 h, median headache score on the sumatriptan day was 4 and median headache score on the placebo day was 6 (Fig. 3). The increase in headache intensity 0-2 h after placebo was significant (*p* < 0.001) while there was no increase after sumatriptan (Fig. 3). The difference between pain intensity difference (PID) 2 h after treatment was significant (*p* = 0.04).

Median headache score 4 h after sumatriptan was 3 and median headache score after placebo was 5 (*p =* 0.017) (Fig. 3). Headache increased 4 h after placebo (*p* = 0.008), but not after sumatriptan (*p* = 0.28) (Fig. 3). PID at 4 h after placebo was significantly larger than after sumatriptan (*p* = 0.0005).

Another primary endpoint, difference in the area under the headache score curve (AUC) 0-4 h after treatment was not significant (*p* = 0.10). Neither was our secondary endpoint, difference in AUC 0-2 h after treatment (*p* = 0.26). Explorative analysis showed that AUC 0-6 h after treatment was significant with a *p*-value of 0.049.

**Fig. 4:**
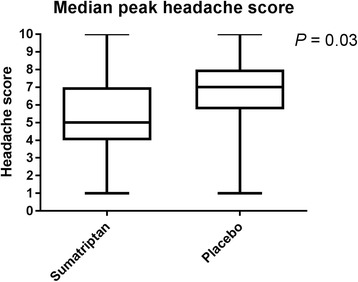
Median peak headache score. Difference in median peak headache score between the two treatment days. Peak headache score was significantly higher on the placebo day compared to the sumatriptan day (*p* = 0.03)

Another secondary end-point, difference in median peak headache score is illustrated in Fig. 4. Median peak headache score was 5 (range 1–10) on the sumatriptan day and 7 (range 1–10) on the placebo day (*p* = 0.03) (Fig. 4).

Rescue medication was taken by 25 patients on the sumatriptan day and by 29 patients on the placebo day. Median time to intake was 7 h and 6.5 h respectively and we do therefore not believe that intake of rescue medication have influenced our data.

### Response of spontaneous attacks to sumatriptan

To test the sensitivity of the study population to sumatriptan, we asked all 30 participants to treat their spontaneous attacks in a double-blind fashion with sumatriptan and placebo. Fifteen patients completed this post-hoc study.

**Fig. 5:**
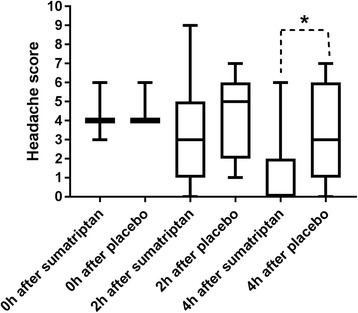
Median headache intensity after treatment of spontaneous migraine attacks. Median headache score at different time points after treatment of spontaneous migraine attacks. Difference in median headache intensity at 2 h: *p* = 0.26. Difference in median headache intensity at 4 h: *p* = 0.006

Our primary end-point, difference in median headache score 2 h after treatment was not significant (*p* = 0.26). At 2 h, median headache score after sumatriptan was 3 and median headache score after placebo was 5. 4 h after treatment the headache score was significantly reduced after sumatriptan (*p* < =0.001), but not after placebo (*p* = 0.32) and the difference between median headache score 4 h after the two treatments (our secondary end-point) was highly significant with a *p*-value of 0.006. At 4 h after treatment, median headache score after sumatriptan was 0 and median headache score after placebo was 3. The difference in median headache score between the two treatment days was also significant at 5 h (*p* = 0.03) and at 6 h after treatment (*p* = 0.04). See Fig. 5 for an overview.

### Explorative analyses

Explorative analyses showed that our results did not differ between sexes, pre-disposed vs. non-predisposed individuals, between patients fulfilling criteria for a migraine-like attack and those who did not, or between patients who did and did not participate in the second study treating their spontaneous attacks.

## Discussion

Cilostazol induced headache in all 30 patients on both provocation days and provoked a migraine-like attack in more than half of the patients. Thus, we confirm previous data showing that cilostazol is a very powerful headache inducing substance ideal for human experimental migraine models [2, 3, 7]. Unfortunately our study did not show a clear cut effect of sumatriptan on the cilostazol induced headache. Therefore this model is not suitable for the testing of novel drugs, with a mechanism of action similar to sumatriptan.

### Was the lack of effect of sumatriptan due to the study population?

Only 15 of the original 30 participants treated their spontaneous attacks. Thus, this study was slightly underpowered. Seven of the original 30 participants were triptan naïve. Only 2 of the 7 treated their spontaneous attacks in a double blind fashion. These 2 patients responded well to sumatriptan. At 2 h after treatment, sumatriptan showed a trend towards efficacy and 4 h after treatment the effect was highly significant. We explain the missing effect at 2 h with the small sample size as it is well known that the effect of triptans is higher at 4 h than at 2 h [8]. The lack of sumatriptan effect on cilostazol headache is therefore unlikely to be due to insensitivity of the study population.

### Can we use the present model to test new acute migraine drugs?

This study represents the third attempt to validate a pragmatic human migraine model by its response to sumatriptan. Ideally an experimental model should respond to all known specific anti-migraine drugs (triptans (5-HT1B/D agonists), CGRP receptor antagonists and 5-HT1F agonists) in double-blind trials. It is unlikely however that such an ideal model would ever be found since mechanisms of migraine are probably heterogenic. Since triptans are most commonly used and seem to perform slightly better than CGRP antagonists and 5-TH1F agonists, we have used sumatriptan as the validating drug for our model [9, 10]. Unfortunately, sumatriptan did not reduce cilostazol induced headache/migraine-like attacks in MO patients but only prevented the headache from developing in intensity. As discussed below, we suggest several explanations for the lack of response. Regardless of explanations, the model proposed in this study cannot be used to test drug candidates that act on the same level of the migraine cascade as sumatriptan. We have previously tested the effect of sumatriptan on cilostazol and isosorbide-5-mononitrate (5-ISMN) induced headache in healthy volunteers, and the results were similar [1, 2]. In a previous study, open and uncontrolled treatment with triptans of cilostazol induced migraine at a later time point showed an apparent positive result. The present study demonstrates the importance of the double-blind design for the validation of drug responds.

### Mode of action: Sumatriptan and cilostazol in the migraine cascade

**Fig. 6:**
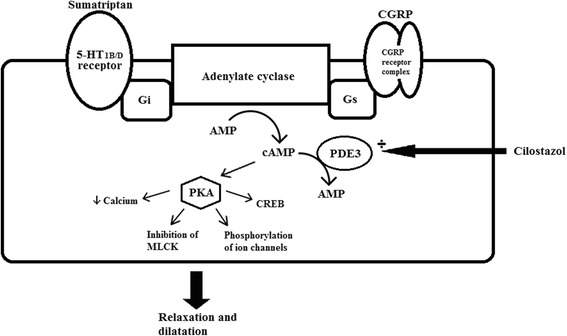
The intracellular mechanisms in a smooth muscle cell after sumatriptan, cilostazol and calcitonin gene related peptide (CGRP) administration. Sumatriptan acts on 5HT1B/D receptors coupled to Gi receptors. This leads to an inhibition of adenylate cyclase and consequently a decrease in cAMP. Cilostazol inhibits PDE3 and thus cause intracellular cAMP accumulation. cAMP activates PKA which leads to a number of intracellular changes and in the end a relaxation and dilatation of the vessel

Sumatriptan exerts its effect at several sites in the migraine cascade. It 1) Causes vasoconstriction via smooth muscle 5-HT_1B_ receptors, 2) Hyperpolarizes afferent trigeminal fibers via 5-HT_1D_ receptors, 3) Inhibits impulse transduction in the trigeminal nucleus via 5-HT_1B/D/F_ receptors, 4) Inhibits CGRP release [11–15]. All these actions happen via activation of 5-HT_1B/D/F_ receptors in the cell membrane. The receptors are coupled to G-proteins which inhibit adenylate cyclase and thereby decrease intracellular cyclic adenosine monophosphate (cAMP) [16–19] (see Fig. 6). Cilostazol is a selective inhibitor of Phosphodiesterase 3 (PDE3) which breaks down cAMP and hence it causes intracellular cAMP accumulation. PDE3 is located in the trigeminal ganglion, vascular smooth muscle cells and endothelium of cerebral and extra cerebral arteries [20, 21].

The present study showed a trend towards efficacy of sumatriptan on cilostazol induced headache in migraine patients. The same trend was also present in healthy volunteers [2] and thus it seems likely that, although small, the effect is real. But sumatriptan cannot overpower the effect of cilostazol. We suggest three likely explanations for that: 1) Sumatriptan causes intracellular changes via an extracellular receptor on the cell membrane whereas cilostazol diffuses into the cell and acts directly on PDE3. In our previous study we used an NO-donor as the headache inducing substance, which also crosses the cell membrane and exerts its effect directly inside the cell [1]. Sumatriptan did not at all reduce that headache. Thus, it seems like the extracellular effect of sumatriptan cannot overpower compounds that intracellularly cause accumulation of second messengers (cAMP and cGMP) [1, 2]. 2) Since there are several pathways in the body leading to increased cAMP, the production is only partly inhibited by sumatriptan and not enough to reverse the increase caused by cilostazol. 3) The half-life of cilostazol is 11 h and thus the migraine inducing effect of cilostazol continued long after the treatment with sumatriptan/placebo.

### The migraine inducing properties of cilostazol

Eighteen (60%) patients developed a migraine-like attack on the sumatriptan day and 19 (63%) on the placebo day. Fourteen patients experienced a migraine-like attack on both provocation days and thus 9 patients did so on only one of the two days. Accordingly, our migraine-induction rate is less than the 86% previously reported [3, 4]. There are several explanations why the induction in our study was less than previously. 1: We gave the patients treatment on both days. 2: Since our treatment was blinded we could not use the otherwise accepted criteria “Headache described as mimicking the patient’s usual migraine attack and treated with acute migraine medication (rescue medication)”. 3: Guo et al. showed that median time to migraine onset after cilostazol was 6 h [3], and Khan et al. [4] reported median 5 h until migraine attack. In our study median time to treatment was 4 h and the induction potential may very well be underestimated, as additional patients may have developed migraine if treatment had been postponed. However, the aim of the study was not to investigate the headache inducing potential of cilostazol and the lower induction rate will not change the outcome of our results.

### Strengths and weaknesses

We calculated that 18 participants were needed and since we enrolled 30 participants, our study was well powered. Our study was placebo-controlled and, as discussed above, treatment efficacy can only be obtained by a double-blind study. Another strength is that we excluded triptan non-responders. A weakness was, however, that we included patients who had no experience with triptans. To compensate, we conducted a follow up study to test whether the spontaneous migraine attacks of the included patients responded to sumatriptan. The patients responded well and thus the lack of sumatriptan response in the provocation study could not be explained by insensitivity of the study population.

## Conclusion

Cilostazol induced headache responds poorly to oral sumatriptan and hence may not be useful for testing novel drugs acting on membrane receptors. The model is, however, useful in studies of migraine mechanisms and may be useful in the testing of novel drugs acting deeper than sumatriptan in the migraine cascade (e.g. intracellularly or directly on ion channels activated by intracellular signaling).

## Additional file

Additional file 1: Datasheet Cilostazol MO. (XLSX 25 kb)

## Abbreviations

5-ISMN: Isosorbide-5-mononitrate
AUC: Area under the curve
cAMP: Cyclic adenylate monophosphate
cGMP: Cyclic guanosine monophosphate
CGRP: Calcitonin gene related peptide
ECG: Electrocardiography
NRS: Numerical rating scale
NSAIDs: Nonsteroidal anti-inflammatory drugs
PDE3: Phosphodiesterase 3
PID: Pain intensity difference
